# Sulforaphene induces apoptosis and inhibits the invasion of esophageal cancer cells through MSK2/CREB/Bcl-2 and cadherin pathway in vivo and in vitro

**DOI:** 10.1186/s12935-019-1061-1

**Published:** 2019-12-19

**Authors:** Chengjuan Zhang, Junxia Zhang, Qiong Wu, Benling Xu, Guoguo Jin, Yan Qiao, Simin Zhao, Yang Yang, Jinwen Shang, Xiaofang Li, Kangdong Liu

**Affiliations:** 1 Biorepository Center, The Affiliated Cancer Hospital of Zhengzhou University, Henan Cancer Hospital, Zhengzhou, Henan People’s Republic of China; 2 Experimental Research Center, Henan University of Chinese Medicine, Zhengzhou, Henan People’s Republic of China; 3 Department of Pathophysiology, School of Basic Medical Sciences, Zhengzhou University, Zhengzhou, Henan People’s Republic of China; 4 China-US (Henan) Hormel Cancer Institute, Zhengzhou, Henan People’s Republic of China; 5 Department of Immunotherapy, The Affiliated Cancer Hospital of Zhengzhou University, Henan Cancer Hospital, Zhengzhou, Henan People’s Republic of China; 6 Laboratory of Bone Tumor, Henan Luoyang Orthopedic Hospital, Zhengzhou, Henan People’s Republic of China; 7 Clinical Systems Biology Research Center, The First Affiliated Hospital of Zhengzhou University, Zhengzhou, Henan People’s Republic of China; 8 Provincial Cooperative Innovation Center for Cancer Chemoprevention, Zhengzhou University, Zhengzhou, Henan People’s Republic of China; 9 Shangqiu Medical College, Shangqiu, Henan People’s Republic of China

**Keywords:** Sulforaphene, Esophageal cancer, Apoptosis, Invasion, MSK2

## Abstract

**Background:**

As a novel type of isothiocyanate derived from radish seeds from cruciferous vegetables, sulforaphene (SFE, 4-methylsufinyl-3-butenyl isothiocyanate) has various important biological effects, such as anti-oxidative and anti-bacterial effects. Recently, sulforaphene has attracted increasing attention for its anti-tumor effects and its ability to suppress the development of multiple tumors through different regulatory mechanisms. However, it has not yet been widely investigated for the treatment of esophageal cancer.

**Methods:**

We observed an increased apoptosis in esophageal cancer cells on sulforaphene treatment through flow cytometry (FCM) analysis and transmission electron microscopy (TEM). Through mass spectrometry (MS) analysis, we further detected global changes in the proteomes and phosphoproteomes of esophageal cancer cells on sulforaphene treatment. The molecular mechanism of sulforaphene was verified by western blot,the effect and mechanism of SFE on esophageal cancer was further verified by patient-derived xenograft mouse model.

**Results:**

We identified multiple cellular processes that were changed after sulforaphene treatment by proteomics. We found that sulforaphene could repress the phosphorylation of CREB through MSK2, leading to suppression of Bcl-2 and further promoted cell apoptosis. Additionally, we confirmed that sulforaphene induces tumor cell apoptosis in mice. Interestingly, we also observed the obvious inhibition of cell migration and invasion caused by sulforaphene treatment by inhibiting the expression of cadherin, indicating the complex effects of sulforaphene on the development of esophageal cancer.

**Conclusions:**

Our data demonstrated that sulforaphene induced cell apoptosis and inhibits the invasion of esophageal cancer through a mechanism involving the inhibition of the MSK2–CREB–Bcl2 and cadherin pathway. Sulforaphene could therefore serve as a promising anti-tumor drug for the treatment of esophageal cancer.

## Background

Esophageal cancer (EC) is one of the most aggressive and common malignancies of the digestive system worldwide and has the 7th highest morbidity rate and 6th highest mortality rate [1, 2]. There are approximately 240,000 new cases of EC in China every year, and the 5-year overall survival rate for EC patients is still less than 25% [3, 4]. EC is categorized into two major histological types: esophageal adenocarcinoma (EAC) and esophageal squamous cell carcinoma (ESCC) [5]. ESCC is the primary histological type of EC and comprises nearly 90% of EC in China [6, 7]. Although several treatments for ESCC have been developed, due to the high invasiveness and frequent regional lymph node metastasis, the prognosis of patients with ESCC is still poor [8, 9]. Recently, targeted therapy for EC showed promise [10, 11]. Novel targeted drugs have been developed and have been shown to have some therapeutic effects [12]. To improve the survival rate of EC patients, novel and effective drugs and treatment strategies are still urgently needed.

China is rich in natural Chinese herbal medicine resources; in fact, traditional Chinese medicine is a precious resource of China. The efficacy of Chinese herbal medicine has not only withstood the scrutiny of long-term medical practice but has also been confirmed by modern scientific research. Sulforaphene (SFE, 4-methylsufinyl-3-butenyl isothiocyanate) is a novel type of isothiocyanate that is derived from radish seeds from cruciferous vegetables [13]. A number of studies have indicated that sulforaphene has multiple biological functions [14, 15]. In adipocytes, sulforaphene could suppress adipogenesis through the hedgehog signaling pathway [14]. Sulforaphene could induce the expression of heme oxygenase (HO-1) and thioredoxin reductase (TrxR) in a dose-dependent manner, thus achieving detoxification effects [16]. Additionally, sulforaphene could eliminate a variety of free radicals, such as hydrogen peroxide and nitrite, and inhibit several bacteria and viruses [17, 18].

Recently, sulforaphene has attracted increasing attention for its anti‐cancer effects in various types of cancers, such as breast cancer, ovarian cancer, hepatocellular carcinoma, and lung cancer [19–22]. Sulforaphene inhibited the development and metastasis of multiple tumors via different regulatory mechanisms [13]. In addition, sulforaphene blocked the progression of lung cancer by targeting the PI3K–AKT pathway and inhibited triple-negative breast cancer (TNBC) through activating the tumor suppressor Egr1 [23, 24]. Sulforaphene has been reported to regulate several signaling pathways involved proliferation, invasion, and apoptosis and has a significant anti-tumor effect, but it has not yet been widely investigated for the treatment of esophageal cancer. Although sulforaphene has significant anti-tumor activity and clinical research value, its potential effects on the growth of esophageal cancer cells and regulatory mechanisms remain unclear.

In this study, we revealed that sulforaphene has the potential to induce the apoptosis and inhibit invision of esophageal cancer cells in vitro and in vivo. Through proteome and phosphoproteome analyses, we identified multiple cellular processes that were changed after sulforaphene treatment. We found that sulforaphene treatment impaired the migration and invasion of cancer cells. Furthermore, sulforaphene could inhibit the expression of MSK2–CREB–Bcl-2 pathway. Meanwhile, we found that sulforaphene promoted tumor cell apoptosis in esophageal cancer in mice. In conclusion, our study revealed that sulforaphene might be a potential therapeutic agent for esophageal cancer.

## Materials and methods

### Antibodies and agents

The following antibodies were used: tubulin (1:1000 dilution, #ab8227, Abcam), MSK2 (1:2000 dilution, #ab99411, Abcam), pCREBs133 (1:1000 dilution, #9198S, CST), and Bcl2 (1:1000 dilution, #15071, CST), Cadherin (1:500 dilution, #ab51034, Abcam).

Sulforaphene was obtained from the Hangzhou Linan Tianhong Technology Co., Ltd., Hangzhou, Zhejiang, China and diluted in ultra-pure water. Sulforaphene was used at concentrations of 0, 5, 10 and 25 μM in vitro, and a dosage of 10 (low dosage) or 50 mg/kg body weight (high dosage) was used in the animal assays.

### Cell apoptosis assay

Approximately 1 × 10^6^ Eca109 cells were treated with DMSO or sulforaphene (1, 2.5, 5, 10, or 25 μM) for 48 h and were then collected, centrifuged, and washed with PBS. Subsequently, the cells were resuspended in 100 μL of binding buffer with 5 μL of annexin V-FITC and incubated at room temperature for 10 min. Subsequently, 5 μL of PI solution was added, and the cells were incubated for another 5 min at room temperature. The apoptotic cells were detected and analyzed with a flow cytometer (Beckman Coulter, Brea, CA).

### Immunohistochemical assay and analysis

To determine the expression levels of the indicated proteins in this study, immunohistochemical (IHC) assays were performed. Briefly, sample sections were fixed with 4% PFA for 30 min and subsequently blocked with 2% BSA for 20 min. The slides were incubated with the indicated antibodies at room temperature for 2 h. Subsequently, the sections were incubated with biotinylated secondary antibody for 1.5 h, and diaminobenzidine was used as a chromogen substrate.

### Cell culture and transfection

EC1 and Eca109 human esophageal cancer cells were purchased from the American Type Culture Collection (ATCC, Manassas, VA). The cells were cultured in DMEM or RPMI-1640 culture medium supplemented with 10% fetal bovine serum (FBS, Gibco, Grand Island, CA, USA) at 37 °C in an incubator with 5% CO_2_.

### Phosphopeptide enrichment and LC/MS–MS measurements

For phosphopeptide enrichment, fractionated peptide mixtures were incubated with an IMAC microsphere suspension by vibration. The IMAC microspheres with enriched phosphopeptides were collected through centrifugation, and the supernatant was subsequently removed. Mass spectrometric detection was performed on an Agilent 1290 LC system (Agilent Technologies) to an Orbitrap Q Exactive Plus mass spectrometer (Thermo Scientific). Peptide segments were dissolved in mobile phase A of liquid chromatography (0.1% (v/v) formic acid aqueous solution) and separated by EASY-nLC 1000 ultra-high performance liquid phase system. The mobile phase A is an aqueous solution containing 0.1% formic acid and 2% acetonitrile, and the mobile phase B is an aqueous solution containing 0.1% formic acid and 90% acetonitrile. The liquid phase gradient was set at 0–48 min, 2–25% B, 48–62 min, 25–40% B, 62–66 min, 40–80% B, 66–70 min, 80% B, and the flow rate was maintained at 400 nL/min., Secondary mass spectrometry data were retrieved using Maxquant.

### Western blot assays

Eca109 cells were lysed to extract the total protein. Then, the samples were analyzed by SDS-PAGE. Subsequently, polyvinylidene fluoride (PVDF) membranes were blocked with 5% milk in TBST buffer and then incubated with primary antibodies targeting MSK2, pCREB, Bcl-2, and tubulin for 1.5 h. Then, the PVDF membranes were incubated with HRP-conjugated secondary antibodies for 1 h. The signals were visualized with an ECL kit. Image Pro software was used to calculate the intensity of the signals in each blot.

### Transmission electron microscopy

Eca109 cells were fixed in phosphate buffer (0.1 M) containing 2.5% glutaraldehyde and 2% formaldehyde (1.5 h), fixed in 1% osmium tetroxide (2 h), dehydrated in ethanol, and embedded in epoxy resin. Then, sections were cut, stained with uranyl acetate and lead citrate, and observed using an H-7500 transmission electron microscope (Hitachi, Tokyo, Japan).

### Wound closure assays

Approximately 3 × 10^5^ esophageal cancer cells Eca109 were plated in 6-well plates and treated with sulforaphene at a concentration of 0, 5, 10, or 25 µM or DMSO and cultured as confluent monolayers. Then, a wound was mechanically generated with a 20-L pipette tip. The cell debris was washed away twice with PBS, and complete culture medium was added to promote wound healing. The wounds were photographed at 0 h, 8 h and 24 h, and the extent of wound closure in the presence of DMSO or sulforaphene treatment was measured and calculated.

### Transwell assays

Esophageal cancer cells Eca109 were treated with DMSO or sulforaphene (concentration) for 48 h and then trypsinized and resuspended in serum-free culture medium. For the migration assays, 1 × 10^5^ cells in 150 µL of culture medium were added to the upper chambers of the inserts (8.0 µm membrane pores; Corning Incorporated) and cultured for 24 h and allowed to migrate toward the bottom chambers, which contained medium with 20% FBS.

For the Matrigel-based Transwell assays, the upper chambers of the filters were coated with 20% Matrigel and incubated at 37 °C for 30 min. A total of approximately 1.5 × 10^5^ cells in 150 µL of culture medium were then added to the upper chambers of the inserts and were allowed to migrate toward the bottom chambers, which contained medium with 20% FBS. Forty hours later, the remaining cells in the top chamber were removed, and the cells on the underside of the chamber were fixed in 4% paraformaldehyde for 20 min and stained with 0.2% crystal violet for 20 min. The quantification of migrated cells was performed by dissolving crystal violet with 10% acetic acid, and the number of cells in each sample was calculated.

### PDX tumor growth assays

Esophageal cancer tissue was collected from a patient diagnosed with moderate esophageal cancer. The project, which aimed to investigate the effect and molecular mechanism of sulforaphene (in humans and animals), was approved by the life science ethics review committee of Zhengzhou University and complied with the ethical requirements of biomedical research issued by international and national regulatory bodies. Written informed consent was obtained from each patient for the current study. The PDX models were initiated by the subcutaneous implantation of esophageal cancer fragments from the patient (approximately 2 mm), which were coated in Matrigel and implanted through subcutaneous flap incisions. All treatment experiments were performed in C. B-17 severe combined immunodeficient mice that were 6 weeks old at the time of PDX injection/implantation. When the tumor volume reached approximately 150 mm^3^, the mice were randomly assigned to groups that were treated with injections of vehicle or sulforaphene. Sulforaphene was freshly prepared before each injection. The first group (10 mice) received 100 μL of vehicle only (2% DMSO and 5% Tween-20 in PBS) every other day for 6 consecutive weeks. The other two groups (10 mice per group) were given 100 μL of sulforaphene (dissolved in 2% DMSO and 5% Tween-20 in PBS) every other day at a dosage of 10 (low dose) or 50 (high dose) mg/kg body weight for 6 consecutive weeks. The tumor volume (length × width × depth × 0.52) was measured, and the body weights were recorded every week. Then, tumors were weighed and fixed in 10% formalin and embedded in paraffin for histological studies.

### Statistical analysis

GraphPad Prism 6.0 software (GraphPad, USA) was used for the statistical analysis. All data in this study were represented as the mean ± standard deviation (SD). Student’s t-test was used for statistical comparisons. The quantifications were based on the results of at least three independent experiments. * Indicates P < 0.05, **P < 0.01 and ***P < 0.001, and P < 0.05 is considered statistically significant.

## Results

### Sulforaphene induces apoptosis and cell cycle arrest in esophageal cancer cells

**Fig. 1:**
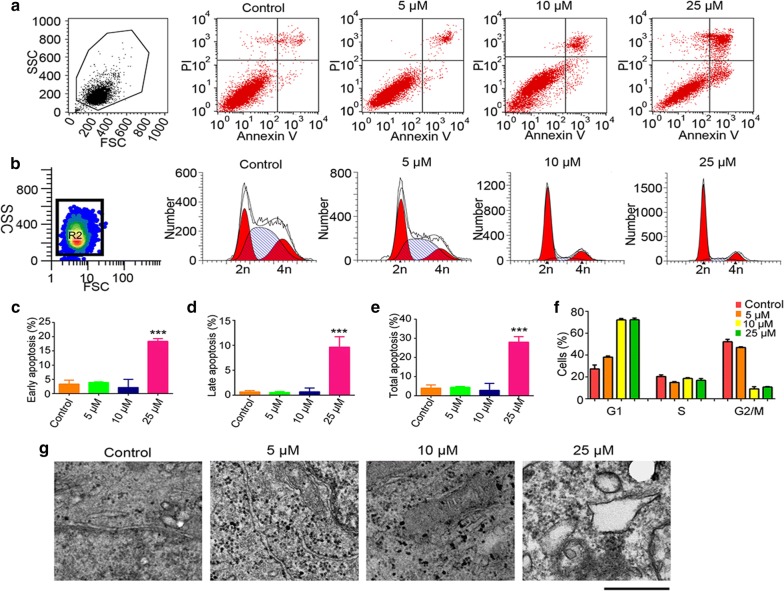
Sulforaphene induces esophageal cancer cell apoptosis. **a** The flow cytometry profile represents the results of annexin V-FITC and PI staining and shows apoptosis in Eca109 cells. **b** The flow cytometry profile represents the results of PI staining and shows the cell cycle of Eca109 cells. **c** Comparison of early apoptosis rates in Eca109 cells with control and sulforaphene treatment. **d** The difference in the late apoptosis rates in Eca109 cells with control and sulforaphene treatment. **e** Comparison of the total apoptosis rates in Eca109 cells with control and sulforaphene treatment. **f** Comparison of the number of cells in the G1, S, and G2/M phases in Eca109 cells with control and sulforaphene treatment. **g** Representative TEM photographs showing cellular surfaces with short microvilli, an amorphous and granular glycocalyx, goblet cells full of mucigen granules and a large number of interdigitations. Data are expressed as the mean SD. * Indicates *P* < 0.05, **P < 0.01 and ***P < 0.001

To investigate the potential of sulforaphene to induce apoptosis in esophageal cancer cells, FCM and TEM experiments were performed. FCM analysis revealed a significant increase in the total population of apoptotic cells in the presence of sulforaphene (Fig. 1a). We also noticed that both the early apoptosis rate and late apoptosis rate of esophageal cancer cells were significantly increased by sulforaphene treatment (Fig. 1c–e). Similarly, a significant increase in cells with an apoptotic phenotype was detected through the observation of Eca109 cells treated with sulforaphene by transmission electron microscopy (TEM), as shown in Fig. 1g. As the sulforaphene concentration increased, we found that the volume of Eca109 cells decreased, the microhairs were shortened, and the chromatin became condensed. Additionally, cyto-membrane dissolution, mitochondrial swelling, and mitochondrial ridge elimination were clearly detected. Through FCM analysis, we also detected changes in the cell cycle after sulforaphene treatment. Interestingly, the results showed a significant increase in cells in the G1 phase in the presence of sulforaphene, indicating the arrest of the cell cycle (Fig. 1b, f).

### Sulforaphene inhibits esophageal cancer cell migration and invasion

**Fig. 2:**
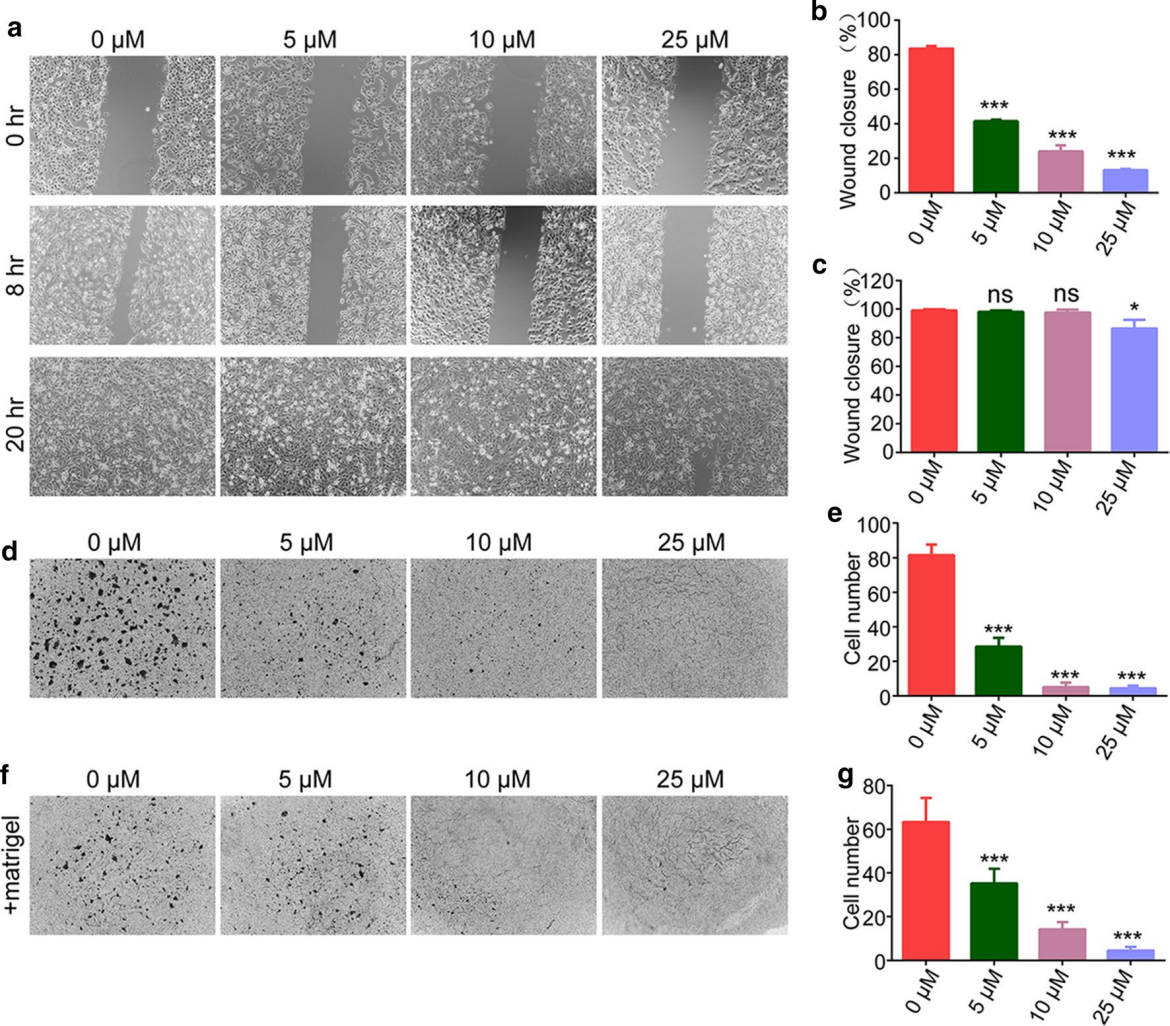
Sulforaphene inhibits the migration and invasion of esophageal cancer cells in vitro. **a**–**c** Wound healing assays were performed using Eca109 cells treated with DMSO or sulforaphene (5, 10, 25 μM), and the percentage of cell migration was measured after 8 or 20 h. **d**, **e** Transwell assays using DMSO- or sulforaphene-treated (5, 10, or 25 μM) Eca109 cells were performed, and the extent of Transwell migration was quantified by counting the cells. **f**, **g** Matrigel-based Transwell assays using DMSO- or sulforaphene-treated (5, 10, 25 μM) Eca109 cells were performed, and the extent of Transwell migration was quantified by cell counting. The results are presented as the mean ± SD

Subsequently, we studied the effects of sulforaphene on the migration and invasion of esophageal cancer cells. Interestingly, treatment with sulforaphene (5, 10, or 25 μM) obviously inhibited the extent of wound closure in Eca109 cells (Fig. 2a–c). Furthermore, in the Transwell assays, Eca109 cells exhibited significantly decreased migration through the membranes that was caused by sulforaphene treatment (5, 10, or 25 μM), and the cell numbers were obviously decreased (Fig. 2d, e). Notably, we detected the invasion capacity of Eca109 cells upon sulforaphene treatment (5, 10, 25 μM) through Matrigel-based transwell assays and found an obvious decrease in the numbers of invading cells (Fig. 2f, g).

### Effects of sulforaphene treatment on differentially expressed proteins and phosphorylated proteins

**Fig. 3:**
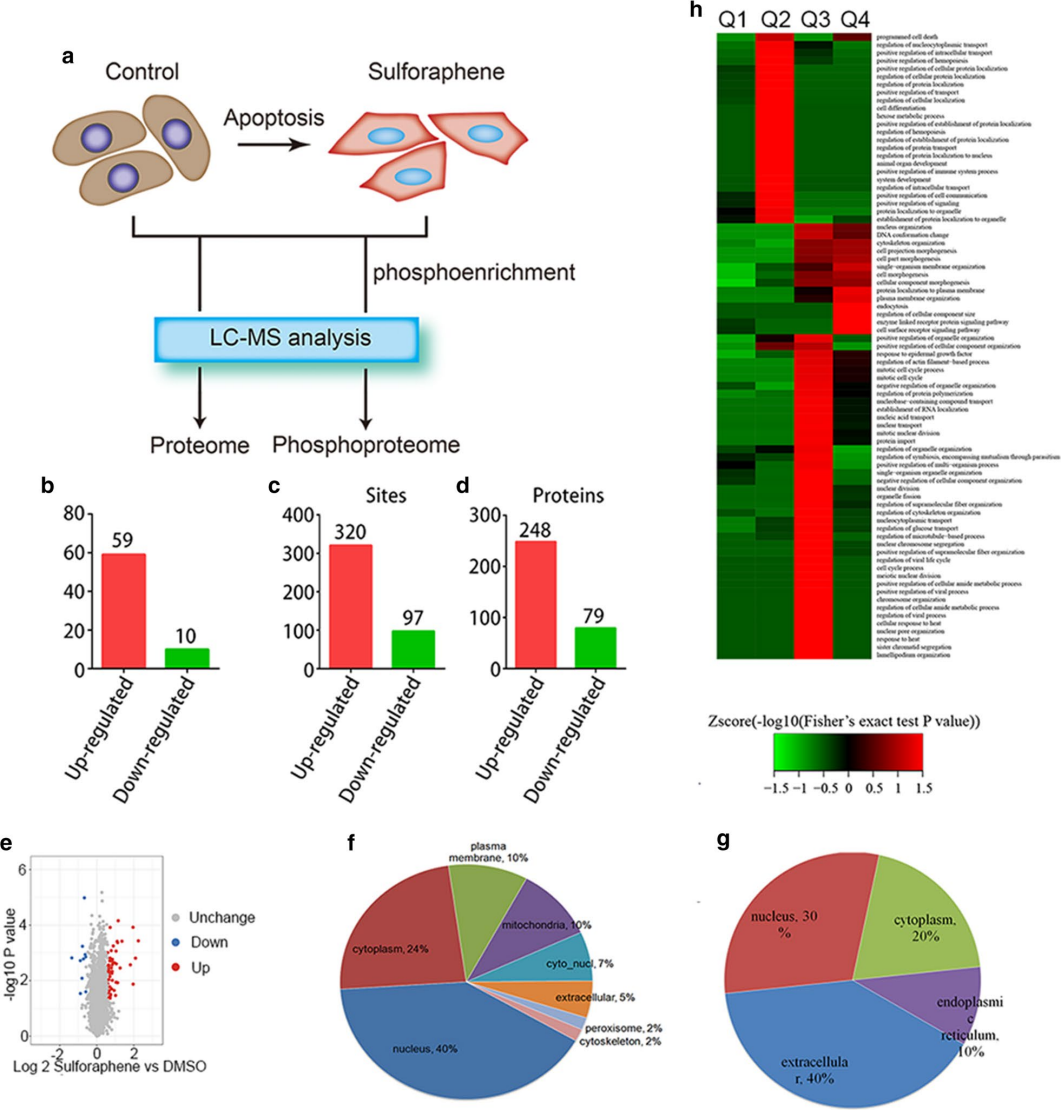
Differentially expressed proteins and phosphorylated proteins induced by sulforaphene treatment. **a** The flow chart of the experiment. Global protein expression and phosphorylation levels in the control and sulforaphene treatment groups were detected by mass spectrometry. **b** Significantly upregulated and downregulated proteins upon sulforaphene treatment were identified through mass spectrometry assays. **c** The number of significantly upregulated and downregulated phosphorylated protein sites upon sulforaphene treatment. **d** The number of significantly upregulated and downregulated phosphorylated proteins upon sulforaphene treatment. **e** Volcano plots of differentially expressed proteins. **f** The cellular distribution of significantly upregulated proteins. **g** The cellular distribution of significantly downregulated proteins. **h** GO analysis heat map of differentially expressed proteins

To gain insight into the mechanisms underlying the promotion of esophageal cancer cell apoptosis by sulforaphene, we performed mass spectrometry (MS) analysis to detect changes in the proteomes and phosphoproteomes of EC1 cells upon sulforaphene treatment, which would provide sufficient data on protein expression and phosphorylation status to identify cellular processes and signaling pathways affected by sulforaphene (Fig. 3a). For the phosphoproteome analysis, phospho-antibody mass spectrometry was used. Global protein expression and phosphorylation level changes in the presence or absence of sulforaphene were examined and analyzed. According to the results of the proteome analysis, 59 significantly upregulated proteins and 10 clearly downregulated proteins were identified for which the fold change was > 1.5 or < 0.667 by comparing DMSO- and sulforaphene-treated cancer cells (Fig. 3b). Similarly, we identified 320 and 97 differentially phosphorylated proteins; 248 significantly upregulated phosphorylated proteins and 79 downregulated proteins in the phosphoproteome were identified (Fig. 3c, d).

Through volcano map analysis, we found modest changes in global gene expression levels after sulforaphene treatment, and several genes were significantly upregulated and downregulated (Fig. 3e). Through localization analysis of differentially expressed genes, we found that the significantly upregulated genes were located in various parts of the cell, including the cytoplasm, nucleus and mitochondria (Fig. 3f). The significantly downregulated genes were mainly found in the cytoplasm, nucleus and endoplasmic reticulum (Fig. 3g). Additionally, a gene expression heatmap and GO analysis revealed that sulforaphene treatment remarkably affected proteins involved in the regulation of several cellular processes (Fig. 3h). Differentially expressed proteins identified from the MS analysis were further analyzed by GO analysis. Several cancer-related biological processes were found to be significantly affected by sulforaphene treatment, such as the cell cycle, cell apoptosis and cell migration (Fig. 3h).

### Sulforaphene could induce apoptosis through MSK2–CREB–Bcl-2 pathway in esophageal cancer cells

**Fig. 4:**
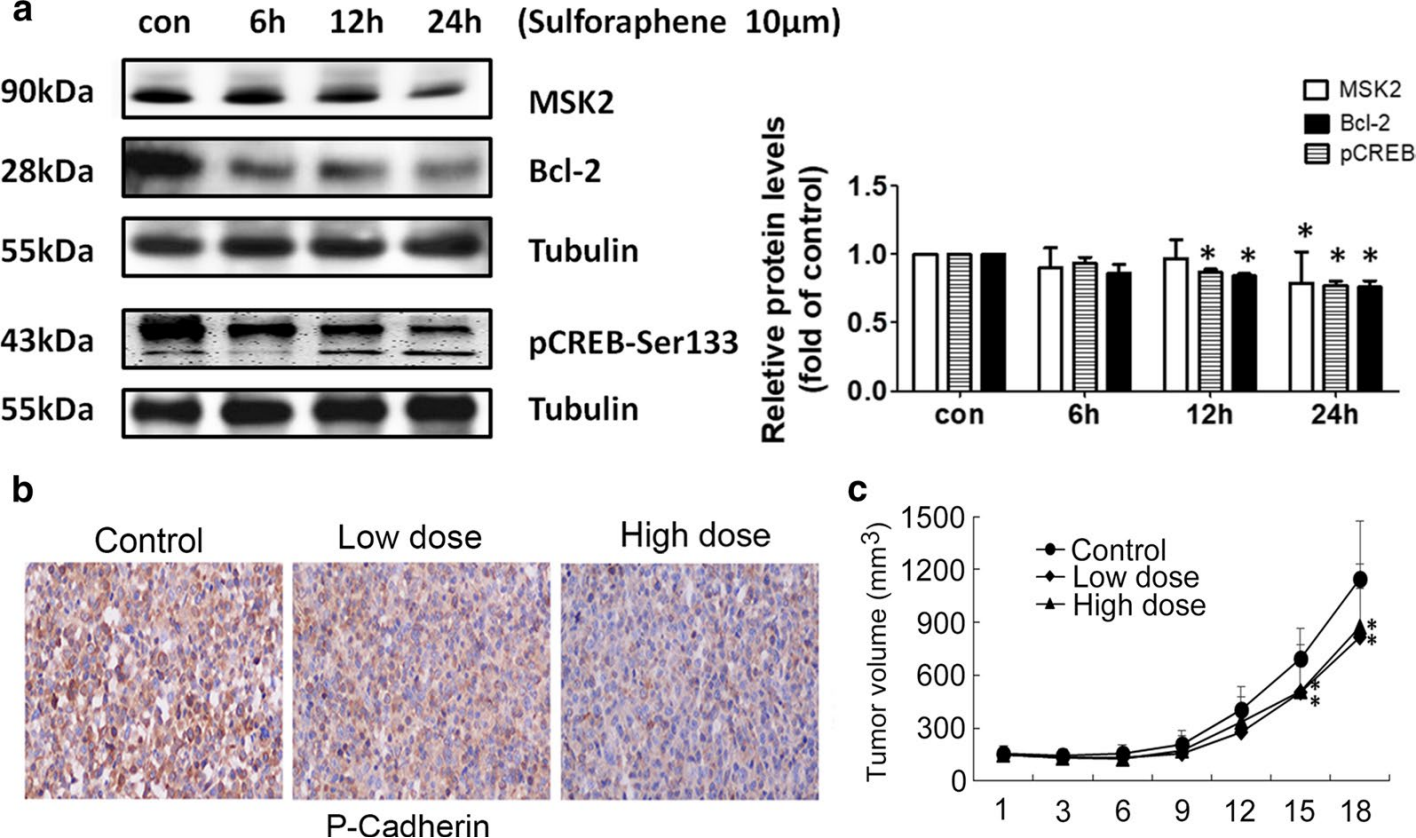
Sulforaphene induce tumor apoptosis through MSK2–CREB–Bcl-2 pathway. **a** Immunoblot analysis of the indicated MSK2, CREB and Bcl-2 expression levels in Eca109 cells with quantification data (n = 3). **b** IHC assays showed the expression levels of Bcl-2, and p-cadherin in both control and sulforaphene-treated tumor tissues isolated from mice. Data are expressed as the mean ± SD. * Indicates *P* < 0.05. **c** Comparisons of tumor volumes in representative mice in the control, low, and high sulforaphene-treated groups

To further explore the molecular mechanism underlying the induction of apoptosis by sulforaphene, the western blot analysis was used and the results revealed that sulforaphene (10 μM) significantly decreased MSK2, pCREB and Bcl-2 protein expression on 6 h, 12 h, 24 h compared with control on 0 h (*P* < 0.05; Fig. 4a). We performed IHC assays to confirm the anti-apoptotic regulatory mechanism of sulforaphene in mice, the results showed the phosphorylated cadherin in tumor tissues from the sulforaphene groups than the control group (Fig. 4b). Then we valuated the effect of sulforaphene on the growth of esophageal cancer patient-derived xenografts (PDX). Tumors were isolated from mice and photographed, and the volumes of the tumors were measured every 3 days. The treatment of mice with sulforaphene reduced the mean tumor volume in the vehicle-treated group faster than that in the sulforaphene-treated (low dosage or high dosage) group (Fig. 4c). However, there were no significant differences in the body weights of mice who received control and sulforaphene treatment. Therefore, these results confirmed that sulforaphene inhibited tumor growth in mice.

## Discussion

**Fig. 5:**
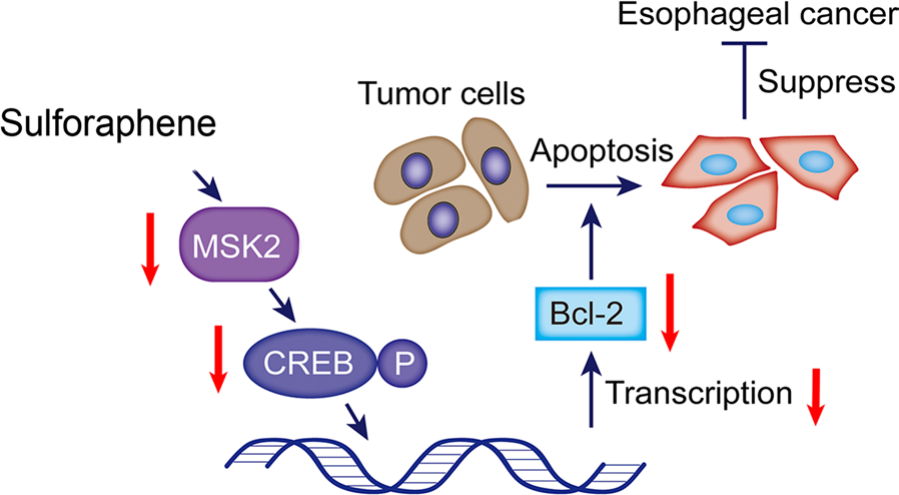
Proposed molecular model of the role of sulforaphene in the esophageal cancer cell. Sulforaphene inhibited the MSK2, and CREB, to restrain the expression of the apoptosis protein Bcl-2, thereby facilitating the apoptosis of esophageal cancer cells and inhibiting cancer development

In this study, we demonstrated that sulforaphene induces apoptosis in esophageal cancer cells. We also showed that sulforaphene treatment is a potential contributor to the inhibition of the MSK2–CREB pathway and then inhibits the expression of anti-apoptotic Bcl-2. (The sequence of experimental procedures are summarized in Fig. 5). This study is an initial step in the exploration of the role of sulforaphene in esophageal cancer cell apoptosis via the MAPK signaling pathway and reveals that sulforaphene may be a promising therapeutic agent for the treatment of esophageal cancer.

Sulforaphene has been found to exhibit anticancer potential against different cancers. It was reported that sulforaphene could decreases human gastric cancer cell viability and induces apoptosis via EGFR, p-ERK1/2 down-regulation pathway [25]. Besides, sulforaphene treatment was also demonstrated to induce G2/M phase cell cycle arrest and apoptosis of colon cancer cells, concomitant with phosphorylation of CDK1 and CDC25B at inhibitory sites [26]. In our study, we also verified that sulforaphene could induce apoptosis in esophageal cancer cells.

Sulforaphene plays important role in apoptosis function in various cancer cell lines but its effect on esophageal squamous cell carcinoma (ESCC) and its mechanism of action remains to be elucidated. MSK2 and its homolog MSK1, are nuclear serine-threonine kinases that belong to the ribosomal protein S6 kinase family [27]. The kinase function of MSKs activated by the upstream MAPKs extracellular signal-regulated kinase (ERK) or p38, then the activation of MSKs by ERK in response to mitogenic signals and by p38 in response to stress [28]. Additionally, the transcriptional effects of MSK2 are mediated by its phosphorylation of transcription factors, such as CREB, ATF1 (activating transcription factor 1), and NF-κB (nuclear factor κB) [29]. Our results showed MSK2 and CREB activity were inhibited by sulforaphene in esophageal squamous cell carcinoma. Bcl-2 is widely known for its anti-apoptotic function, and the repression of Bcl-2 expression is an available method for cancer therapy [30]. Notably, our results verified that CREB is involved in sulforaphene-induced apoptosis through down regulation of Bcl-2 expression, which therefore mediated the induction of cell apoptosis.

Besides the MAPK signaling pathway, other pathways and mechanisms mediate the occurrence and development of multiple types of tumors induced by sulforaphene. An increasing number of studies have demonstrated that sulforaphene promotes tumorigenesis and metastasis through the regulation of cell proliferation, migration, invasion, and apoptosis [21]. Recently, sulforaphene was reported to inhibit tumor growth in colon cancer through the induction of glutathione ablation and microtubule depolymerization [26]. Sulforaphene suppressed lung tumorigenesis by targeting the PI3K–AKT signaling pathway and enhanced the radiosensitivity of hepatocellular carcinoma via the inhibition of the NF-κB signaling pathway, which could also result in the apoptosis of HCC cells [21, 23]. Interestingly, sulforaphene induces mitophagic cell death via p62/SQSTM1 accumulation and AMPK inhibition [15].

Here, we found that sulforaphene could dramatically induce apoptosis and cell cycle arrest in esophageal cancer cells and effectively inhibit tumor growth in mice. Similarly, our previous study confirmed that a promising agent, HOI-02, facilitated apoptosis and cell cycle arrest in ESCC through ROS. Additionally, a previous study indicated that sulforaphene caused cytotoxicity and promoted the apoptosis of human hepatocarcinoma HepG2 cells via increases in caspase 3 and 9 activity [31]. Sulforaphene could also promote human gastric cancer cell apoptosis by downregulating EGFR and p-ERK1/2 and inhibiting the MAPK signaling pathway [25, 32]. In addition, another study demonstrated that a combination of sulforaphene and carboplatin could effectively inhibit proliferation and induce apoptosis in human NSCLC A549 cells through cell cycle arrest, caspase inhibition and mitochondrial membrane potential disruption [22]. Notably, our findings revealed the molecular mechanism underlying the stimulation of esophageal cancer cell apoptosis by sulforaphene. However, previous studies reported that apoptosis and senescence phenomena may occur when stress happened to cells. It also demonstrated that some cells are prone to senescence rather than apoptosis after low doses of radiation [33]. Therefore, in the next experiment, we will continue to study whether sulforaphene have the effect on senescence of esophageal cancer cells.

Tumor metastasis is induced by the invasion of tumor cells into blood vessels, and P-cadherin has been widely reported to be involved in this process. In this study, we confirmed that sulforaphene inhibited cell migration and invasion in esophageal cancer through the inhibition of P-cadherin expression. Similarly, a previous study revealed that P-cadherin inhibited the suppression of invasion by E-cadherin through the disruption of E-cadherin/p120-cadherin complexes near the cell cortex. We next need to confirm whether sulforaphene inhibits esophageal cancer cell migration through a similar regulatory mechanism.

## Conclusion

Our findings, together with those of other studies, indicated that sulforaphene could repress MSK2, and CREB, Bcl-2, down-regulate the C-cadherin expression and thereby induced apoptosis and inhibited invasion in esophageal cancer cells. We would next investigated whether sulforaphene could inhibit the proliferation of esophageal cancer cells through these reported signaling pathways according to other studies with sulforaphene. To be concluded, sulforaphene could have the potential to serve as an anti-tumor drug through the promotion of tumor cell apoptosis and the inhibition of cell invasion. Which could be effectively used in the clinical treatment of esophageal cancer.

## Data Availability

The datasets supporting the conclusions of this study are included in this article. Any requests for data or materials can be sent to the corresponding author.

## Abbreviations

ESCC: esophageal squamous cell carcinoma
FCM: flow cytometry
TEM: transmission electron microscopy
MS: mass spectrometry
DMSO: dimethylsulfoxide
CREB: cAMP-response element binding protein
PDX: patient-derived tumor xenograft
